# 1171. Measles and Rubella Seroprevalence among HIV-infected and uninfected Children and Adults in Zambia

**DOI:** 10.1093/ofid/ofab466.1364

**Published:** 2021-12-04

**Authors:** Yangyupei Yang, Simon Mutembo, Andrea Carcelen, Kyla Hayford, Francis Mwansa, William J Moss

**Affiliations:** 1 Johns Hopkins Bloomberg School of Public Health, Baltimore, Maryland; 2 Ministry of Health Zambia, Choma, Southern, Zambia

## Abstract

**Background:**

Despite the availability of safe and effective vaccines, measles and congenital rubella syndrome remain important causes of morbidity and mortality. HIV-infected individuals may be more vulnerable to measles because of poor immune responses to vaccination. Population-level estimates and comparisons of measles and rubella seroprevalence between HIV-infected and uninfected children and adults in sub-Saharan Africa are needed to guide vaccination policy and control strategies.

**Methods:**

This cross-sectional study was performed by analysing a selected and weighted subsample from the Zambia Population HIV Impact Assessment survey (ZAMPHIA). ZAMPHIA was conducted in 2016 to estimate national HIV incidence and prevalence in Zambia. Dried blood spots and plasma samples were tested for IgG antibodies to measles and rubella viruses using a commercial enzyme immunoassay. We estimated national age-specific measles and rubella seroprevalence by HIV infection status using hierarchical generalized additive models.

**Results:**

Specimens from 9521 HIV-uninfected (3840 children age under 10 years, 3981 youth age 10-19 years, and 1700 adults age 20-49 years) and 331 HIV-infected (53, 107, and 171 respectively) individuals were included in the study. Measles seroprevalence was lower among HIV-infected children (46.4%) compared to HIV-uninfected children (76.4%, p < 0.001). In both HIV-uninfected and HIV-infected individuals, measles seroprevalence increased steadily with age but more rapidly in the HIV-infected until about the age of 20 years when the seroprevalence was similar between the two groups. Above 20 years, measles seroprevalence was similar between HIV-infected and uninfected adults. There was no significant difference in rubella seroprevalence between HIV-infected and HIV-uninfected individuals.

Figure 1. Measles and Rubella Age-specific Seroprevalence

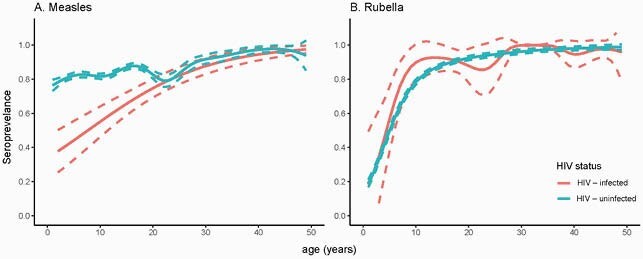

The lines represent generalized additive model fits for the mean (solid) and 95% confidence intervals (dashed). Data are grouped by age in years and year 0 includes only specimens from children 9-11 months. Rubella-containing vaccine was not available in the public sector prior to the serosurvey.

**Conclusion:**

Measles seroprevalence was lower among HIV-infected than uninfected children and youth. HIV-infected children would likely benefit from revaccination. Many children were susceptible to rubella before the introduction of the combined measles and rubella vaccine in Zambia.

**Disclosures:**

**Kyla Hayford, PhD, MA**, **Pfizer, Inc.** (Other Financial or Material Support, KH conducted the study and analyses while working at the Johns Hopkins School of Public Health but is an employee at Pfizer, Inc. as of 26 October 2020.)

